# *In-situ* synthesized perovskite/polyhedral oligomeric silsesquioxane nanocomposites for robust X-ray imaging

**DOI:** 10.1016/j.isci.2024.110951

**Published:** 2024-09-14

**Authors:** Hai Liang, Fan Wu, Runan Xia, Wei Wu, Siqi Li, Panpan Di, Miao Yang

**Affiliations:** 1 Department of Pharmacy, The People’s Hospital of Bozhou, Bozhou, Anhui Province, P.R. China; 2 School of Physics and Optoelectronic Engineering, Anhui University, Hefei 230601, Anhui, P.R. China

**Keywords:** Applied sciences, Materials synthesis

## Abstract

Perovskites are extensively studied in scintillation detection due to their low cost, simple synthesis, high scintillation light yield, and rapid decay times. However, their instability to light and radiation leads to scintillation performance degradation. To address these stability concerns, this paper proposes a new perovskite nanocrystal (NC) synthesis method that employs aminopropyllsobutyl polyhedral oligomeric silsesquioxane (POSS) as a ligand and a coating layer to passivate the perovskite NCs, significantly enhancing their stability and photoluminescence efficiency. Furthermore, the resultant perovskite/aminopropyllsobutyl POSS nanocomposites exhibit remarkable capabilities in X-ray detection limits, imaging quality, and radiation hardness. These findings underscore the potential of enhanced perovskite in revolutionizing the field of scintillator materials, offering promising pathways for their future applications and development.

## Highlights

• A co-precipitation method to synthesize CsPbBr_3_/NH_2_-POSS composite is proposed
• The CsPbBr_3_ nanocrystals demonstrate enhanced stability through POSS encapsulation
• The composite material features high radiation resistance and low detection limits

## Graphical abstract

**Figure:**
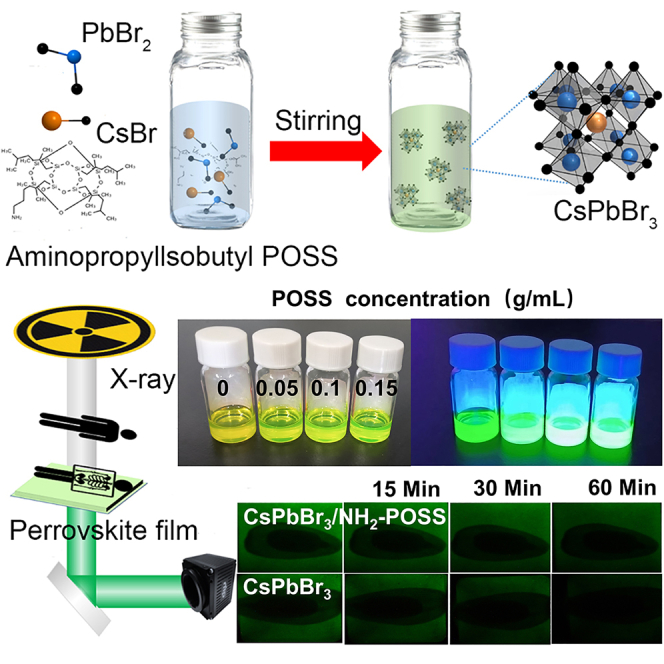

## Introduction

Perovskite materials have attracted significant attention in the field of optoelectronics, particularly as scintillators, due to their several intrinsic advantages.^1^^,^^2^^,^^3^^,^^4^ These materials are distinguished by their cost-effectiveness, straightforward synthesis, and notably high luminesce efficiency.^5^^,^^6^^,^^7^ Moreover, perovskites exhibit rapid decay times, ensuring quick responses in high-speed scintillation detection applications.^8^^,^^9^ Another particularly beneficial feature of perovskites is their structural versatility, which allows for precise modification of their properties through the modification of constituent elements or the incorporation of various dopants, such as rare-earth elements, to enhance specific functionalities.^10^^,^^11^

Despite these advantageous properties, perovskites are intrinsically easily decomposed due to their ionic structure, presenting a significant obstacle for their application in scintillator detectors.^2^^,^^12^^,^^13^ This decomposition is aggravated by prolonged exposure to light and radioactive radiation, resulting in a gradual degradation known as photolytic or radiation degradation. Such degradation adversely impacts the light output efficiency and scintillation performance.^14^ Additionally, perovskites demonstrate sensitivity to moisture and thermal instability, which further lead to their decomposition.^15^^,^^16^ During the synthesis process, defects that may exist in the perovskite structure, such as lattice defects, can also affect the performance and stability during long-term use.^6^^,^^13^^,^^17^

To address these instability challenges, various strategies have been devised aimed at enhancing the stability of perovskite scintillators.^5^^,^^18^ These strategies involve optimizing the composition of organic and inorganic components to augment chemical stability,^19^ implementing advanced crystal engineering and microstructure control techniques to bolster both physical and chemical robustness and utilizing diverse doping techniques to increase thermal stability and radiation resistance.^20^ Wang et al. and others have *in situ* synthesized Ruddlesden-Popper-type perovskite nanocrystals (NCs) in mesoporous silica, showing good stability and luminescence efficiency. These NCs have been utilized to fabricate flexible scintillation detection screens capable of achieving resolutions as high as 14 lp/mm.^21^ Zaffalon et al. have significantly improved the radiation hardness of CsPbBr_3_ NCs through post-synthesis fluorination, making them resistant to γ-ray radiation doses of up to 1 MGy.^22^

Here, we propose a novel rapid preparation method for perovskite NCs encapsulated in polyhedral oligomeric silsesquioxane (POSS): specifically, aminopropyllsobutyl POSS. This method involves initially dissolving the POSS in a toluene solution, followed by the addition of perovskite precursors. The concentration of aminopropyllsobutyl POSS can moderate the growth rate of the NCs. The resultant formation is a composite structure (perovskite/aminopropyllsobutyl POSS), wherein amino groups form stable bonds with the lead (Pb) ions within the perovskite lattice. The presence of the POSS-NH_2_ attribute enhanced stability and elevated photoluminescence (PL) efficiency of the NCs. When evaluated as scintillator detectors, these composite materials demonstrated significant performance, achieving a substantial light yield of 23,660 photons MeV^−1^ and exhibiting high radiation resistance, which underscores their potential in advanced optoelectronic and radiation detection applications.

## Results

### Synthesis of CsPbBr_3_/aminopropyllsobutyl POSS

We begin by contrasting our synthesis approach with a co-precipitation method for fabricating CsPbBr_3_/aminopropyllsobutyl POSS nanocomposites.^23^^,^^24^^,^^25^ Using the traditional co-precipitation technique, a solution of CsBr and PbBr_2_ in N,N-Dimethylformamide (DMF) is pre-heated to 85°C and then injected into a toluene solution. The initially clear toluene solution promptly transitions to a yellow-green color. Ultimately, the solution displays a deeper yellow color with reduced light transmission, indicating particle inhomogeneity. Transmission electron microscopy (TEM) analysis further confirms an uneven distribution of perovskite NCs sizes, illustrating the challenges in controlling the growth of NCs via this rapid synthesis method (Figure S1).

**Figure 1:**
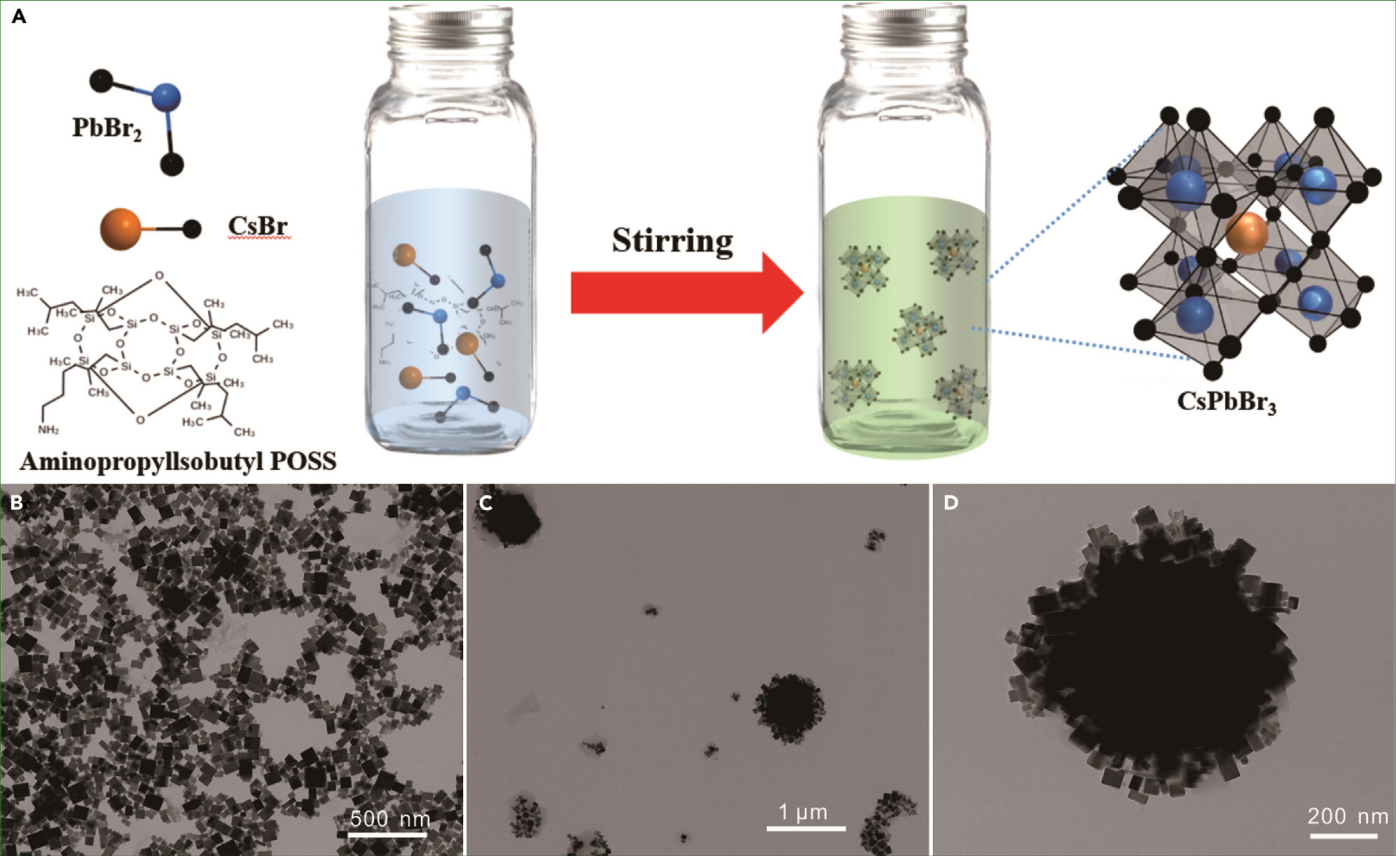
Synthesis of CsPbBr_3_/aminopropyllsobutyl POSS and its TEM characterization (A) Schematic diagram illustrating the synthesis of CsPbBr_3_/aminopropyllsobutyl POSS nanocomposite NCs. (B) TEM characterization of perovskite NCs obtained by the co-precipitation method. (C and D) TEM characterization of CsPbBr_3_/aminopropyllsobutyl POSS nanocomposite NCs.

Conversely, in our method using aminopropyllsobutyl POSS as a ligand and a coating layer, the growth of perovskite is significantly decelerated as the concentration of aminopropyllsobutyl POSS increases. For instance, at a concentration of 0.15 g/mL of aminopropyllsobutyl POSS pre-dissolved before, the toluene solution color did not change immediately after the injection of perovskite precursors (Figure S2). Approximately 5 min later, the solution shifts to a light white and emits a faint green light under UV exposure. Over a period from 10 to 80 min, the color intensifies from light green to green, accompanied by a pronounced green luminescence. TEM analysis shows that the perovskite NCs are effectively encapsulated by aminopropyllsobutyl POSS, indicating improved control over the NCs growth (Figures 1C, 1D, and S3).

### Optical properties of CsPbBr_3_ and CsPbBr_3_/aminopropyllsobutyl POSS

**Figure 2:**
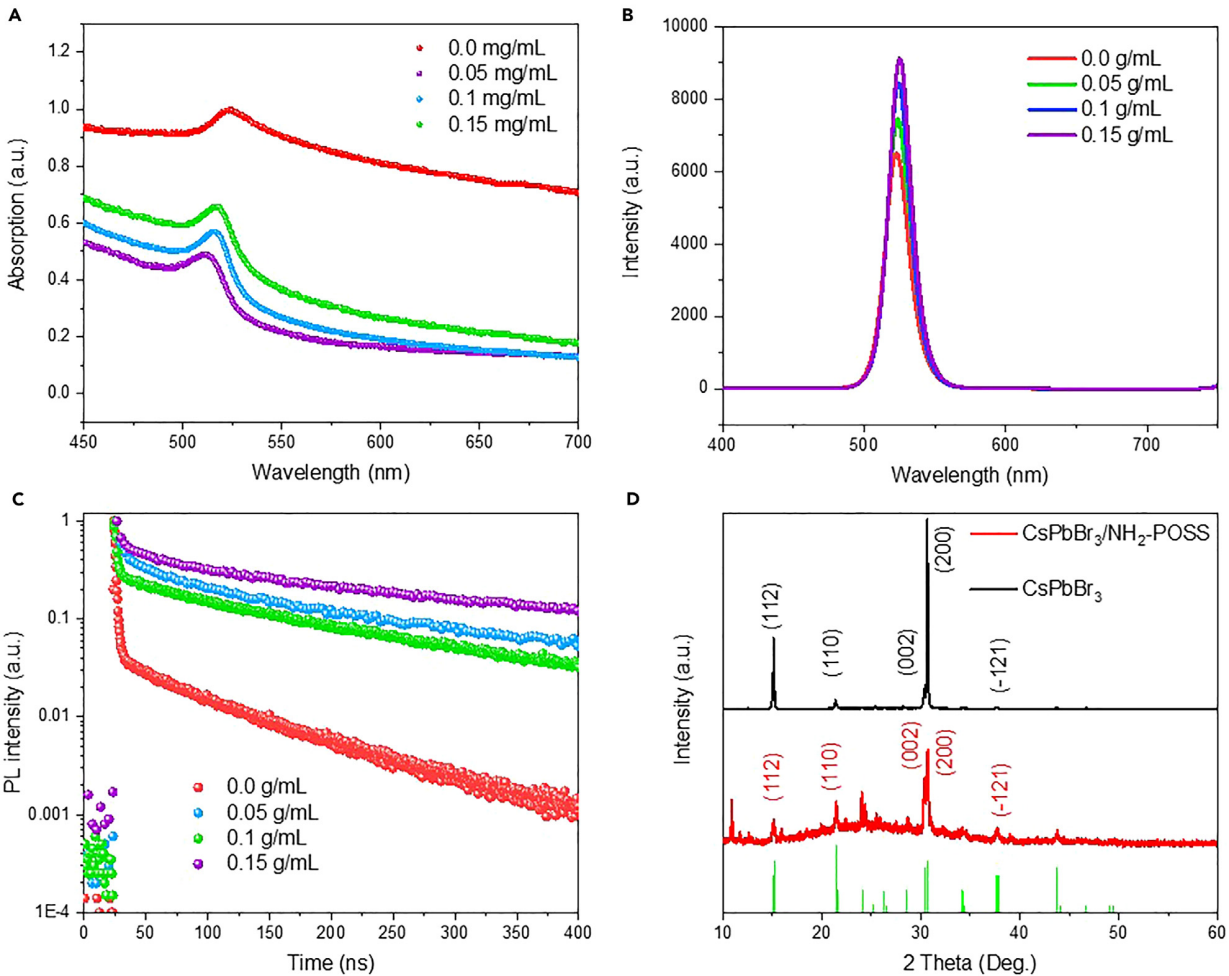
Optical properties and structural analysis of CsPbBr_3_/aminopropyllsobutyl POSS (A) The UV-visible spectra of perovskite NCs synthesized with varying concentrations of aminopropyllsobutyl POSS in toluene. (B and C) The PL spectra and corresponding decay lifetime curves of the NCs, indicating the effects of POSS concentration on the optical properties. (D) The XRD patterns of CsPbBr_3_ and CsPbBr_3_/aminopropyllsobutyl POSS nanocomposites, both aligning with the monoclinic phase of CsPbBr_3_ as indicated by the reference PDF #18-0364.

Figure 2A shows the absorption spectra of perovskite NCs synthesized with different mass fractions of aminopropyllsobutyl POSS in a toluene solution. It can be observed that the CsPbBr_3_ NCs obtained solely by the co-precipitation method exhibit more pronounced scattering with an absorption peak around 515 nm. In contrast, the absorption peak at 515 nm of CsPbBr_3_/aminopropyllsobutyl POSS nanocomposites is more prominent. Under the same excitation power, the perovskite NCs synthesized with 0.15 g/mL aminopropyllsobutyl POSS in the original solution show the brightest PL intensity. Figure 2C presents the corresponding lifetime curves, which can be well fitted by a two-exponential-decay function $I\left(t\right)=I_{0}+A_{1}\;\exp\left(-\frac{t}{\tau_{1}}\right)+A_{2}\;\exp\left(-\frac{t}{\tau_{2}}\right)$, where $\tau_{1}$ and $\tau_{2}$ are the two characteristic lifetimes and $A_{1}$ and $A_{2}$ are their respective coefficients. The total lifetime is $\tau =\left(A_{1}{\tau_{1}}^{2}+A_{2}{\tau_{2}}^{2}\right)/\left(A_{1}\tau_{1}+A_{2}\tau_{2}\right)$. The lifetimes of the as-prepared samples with different aminopropyllsobutyl POSS are listed in Table 1. It is shown that the calculated lifetime of CsPbBr_3_/aminopropyllsobutyl POSS nanocomposites was ranging from 37.51 to 21.15 ns, ensuring the CsPbBr_3_/aminopropyllsobutyl POSS nanocomposites serve as a fast fluorescent response in scintillation detection.

**Table 1:** The fitting parameter of time-resolved PL decay

	A_1_	τ_1_	A_2_	τ_2_	τ
0.15 g/mL POSS	0.87	37.73	0.13	1.49	37.51
0.1 g/mL POSS	0.77	33.33	0.23	1.26	32.96
0.5 g/mL POSS	0.68	28.03	0.32	0.87	27.64
0	0.61	21.66	0.39	0.84	21.15

Figure 2D displays the X-ray diffraction (XRD) spectra of both CsPbBr_3_ NCs and CsPbBr_3_/aminopropyllsobutyl POSS nanocomposites, both corresponding to the monoclinic phase of perovskite (PDF #18-0364),^26^^,^^27^ indicating that the preliminary addition of aminopropyllsobutyl POSS does not alter the crystal phase of the perovskite NCs. The method of using aminopropyllsobutyl POSS as a coating layer can effectively improve the stability of the synthesized perovskite NCs. After long-term storage comparison, it was found that the perovskite toluene solution without the addition of aminopropyllsobutyl POSS quickly decomposed after long-term storage, and the color of the solution changed from yellow green to light yellow. The luminescence intensity was also significantly reduced. In contrast, the CsPbBr_3_/aminopropyllsobutyl POSS toluene solution still possesses strong fluorescence emission after long-term storage (Figures S4–S7). More interestingly, this method can be prepared on a large scale, providing the possibility for practical engineering applications (Figure S8).

**Figure 3:**
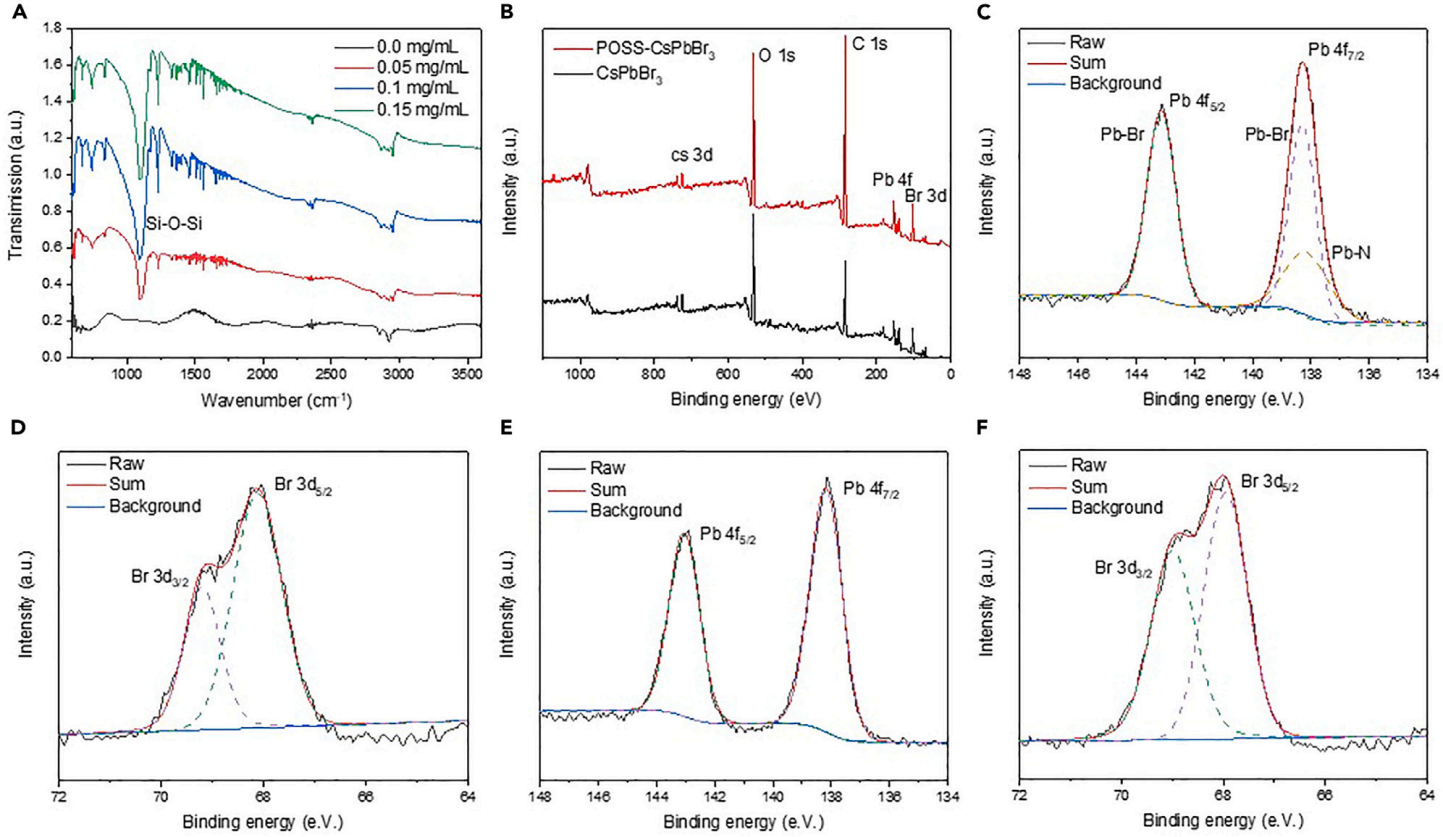
Analysis of bonding characteristics between perovskite and aminopropyllsobutyl POSS (A) Fourier transform infrared spectra of perovskite NCs synthesized with varying mass fractions of aminopropyllsobutyl POSS. (B–F) (B) Full-scan XPS spectra and (C–F) high-resolution XPS of Pb and Br in CsPbBr_3_/aminopropyllsobutyl POSS and CsPbBr_3_ NCs.

In the infrared spectrum shown in Figure 3A, compared to pure CsPbBr_3_ NCs, the infrared spectrum of CsPbBr_3_/aminopropyllsobutyl POSS nanocomposites displays a wavenumber at 1,100 cm^−1^. Figures 3B–3F shows the XPS results, which depict the change in binding energy according to the doping element. The XPS spectrum reveals signals of Cs, Pb, Br, and N elements in both CsPbBr_3_ NCs and CsPbBr_3_/aminopropyllsobutyl POSS, with the corresponding high-resolution XPS spectrum displaying typical Pb and Br signals.^28^^,^^29^ The high-resolution spectrum of Pb 4f was compared for the CsPbBr_3_ NCs and CsPbBr_3_/aminopropyllsobutyl POSS, as shown in Figures 3C and 3D. Detailed analysis of the Pb XPS data reveals the presence of additional Pb-N bonding, indicating that the aminopropyllsobutyl POSS surface provides additional amino groups to bond with Pb.^30^

### Scintillation measurement of CsPbBr_3_/aminopropyllsobutyl POSS

**Figure 4:**
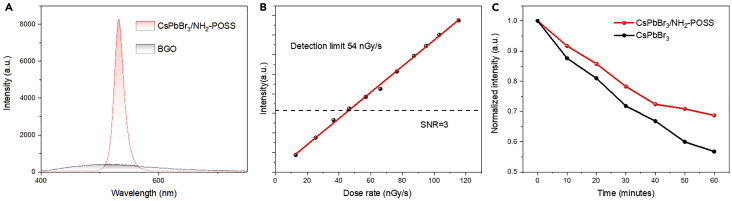
Scintillation properties of CsPbBr_3_/aminopropyllsobutyl POSS (A) Comparison of the RL of POSS-CsPbBr_3_ composite films with commercial BGO crystals. (B) RL intensity and detection limits of POSS-CsPbBr_3_ composite films under different X-ray dose rates. (C) Comparison of radiation stability of POSS-CsPbBr_3_ and CsPbBr_3_ under the same X-ray dosage.

Subsequently, we examined the scintillation performance of CsPbBr_3_/aminopropyllsobutyl POSS. It was found that the absolute light output of the CsPbBr_3_/aminopropyllsobutyl POSS scintillator is about 23,660 photons MeV^−1^ (Figure 4A), approximately 2.6 times that of bismuth germanate crystal (BGO) (The light output is approximately 9,000 photons MeV^−1^). The minimum detection of CsPbBr_3_/aminopropyllsobutyl POSS is 54 nGy/s. Under application-level irradiation, sufficient radiation hardness is crucial. Herein, we stetted a radiation dose of 8.89 μGy/s and conducted a continuous 60-min test on the radioluminescence (RL) stability of the CsPbBr_3_/aminopropyllsobutyl POSS and CsPbBr_3_ film (Figures 4C and S10). The RL intensity of CsPbBr_3_/aminopropyllsobutyl POSS did not decrease as significantly as that of pure CsPbBr_3_, demonstrating the enhanced radiation resistance of CsPbBr_3_ provided by the POSS encapsulation.

### X-ray imaging of CsPbBr_3_/aminopropyllsobutyl POSS

**Figure 5:**
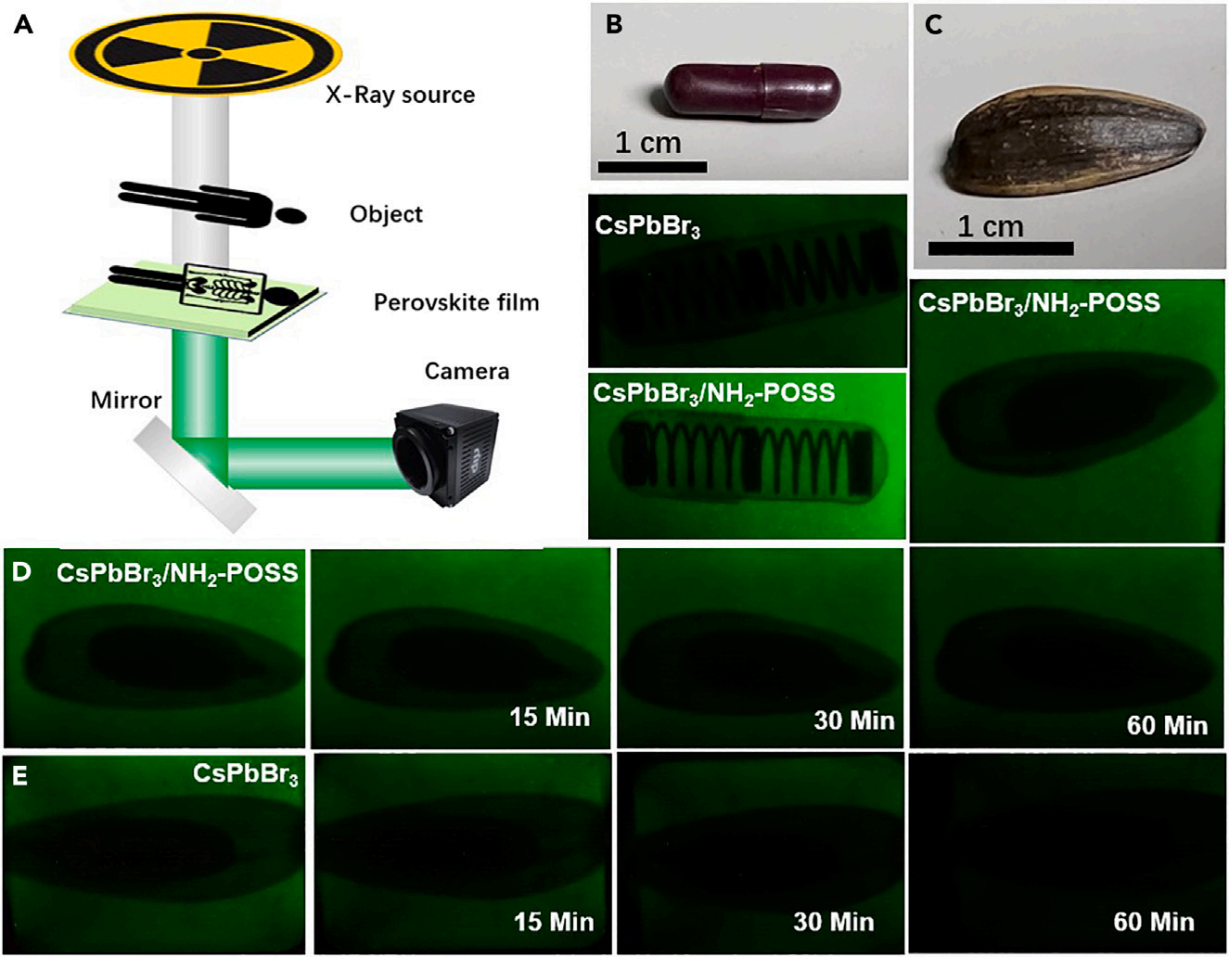
X-ray imaging comparison of CsPbBr_3_/aminopropyllsobutyl POSS and CsPbBr_3_ NCs (A) Schematic of radiation imaging, (B and C) X-ray images of a capsule (containing a spring) and sunflower seeds using CsPbBr_3_/aminopropyllsobutyl POSS as the scintillation screen, and (D and E) comparison of long-term X-ray imaging between CsPbBr_3_/aminopropyllsobutyl POSS and CsPbBr_3_.

To further validate the feasibility and potential of CsPbBr_3_/aminopropyllsobutyl POSS nanocomposite material for practical X-ray applications, we constructed a custom-designed optical imaging system, as illustrated in Figure 5. The samples were placed between the X-ray source and the CsPbBr_3_/aminopropyllsobutyl POSS nanocomposite film. The charge-coupled device (CCD) camera captured images from the rear of the scintillation screen. A mirror was placed to reflect light rays to prevent radiation interference with the CCD device, averting noise and protecting the CCD camera. Due to the different X-ray absorption capabilities of the tested object, the X-ray intensity reaching the perovskite scintillation screen varies. Subsequently, the perovskite scintillation screen converts the X-ray into visible light, promoting clear optical imaging. As depicted in Figure 5B, X-ray imaging enables the clear visualization of internal structures, such as a spring-contained capsule, wherein the inner spring exhibits greater X-ray attenuation than the outer capsule material, rendering the invisible spring discernible. By using the X-ray test scale (tape 39) for direct observation, the estimated X-ray imaging resolution for CsPbBr_3_ NWs/PM597 scintillator is higher than 10 lp mm^−1^ (Figure S11).

The remarkable resolution ensures clear imaging of sunflower seed interiors even when there exists minimal disparity in X-ray attenuation capabilities of the object (Figure 5D). Moreover, the CsPbBr_3_/aminopropyllsobutyl POSS nanocomposite scintillator outperforms in imaging during prolonged exposure to high-dose irradiation. Figure 5E illustrates the behavior of the CsPbBr_3_/aminopropyllsobutyl POSS nanocomposite scintillator film and CsPbBr_3_ scintillator film under a radiation dose of 2 Gy/s. Evidently, The CsPbBr_3_ scintillator film is more significantly damaged by accumulated X-rays, whereas the CsPbBr_3_/aminopropyllsobutyl POSS nanocomposite scintillator film maintains imaging clarity even after continuous exposure for an hour, thus verifying the ability of aminopropyllsobutyl POSS in enhancing the radiation hardness of perovskites.

### Conclusion

In summary, we have developed a method for synthesizing perovskite NCs using amine-modified POSS for passivation. This technique capitalizes on the amine passivation effect on the POSS surface, resulting in CsPbBr_3_/aminopropyllsobutyl POSS composite NCs that exhibit significantly enhanced luminescence efficiency compared to standard CsPbBr_3_ NCs. This method also demonstrates excellent control over particle size and morphology of the perovskite NCs. Furthermore, due to the protection of aminopropyllsobutyl POSS, the perovskite overcomes its stability shortcomings. Additionally, the CsPbBr_3_/aminopropyllsobutyl POSS nanocomposite scintillator film not only surpasses commercial BGO crystals in light yield but also features a low detection limit of 54 nGy/s and excellent spatial resolution. The CsPbBr_3_/aminopropyllsobutyl POSS nanocomposites also have demonstrated radiation hardness under radiation dose. This approach not only improves the performance of scintillator films but also opens up new avenues for developing advanced detection technologies with higher sensitivity and durability in radiation environments. Our work provides valuable insights and a promising strategy for enhancing the stability and efficiency of perovskite scintillators, potentially revolutionizing their application in X-ray imaging and other high-performance optoelectronic devices.

### Limitations of the study

Due to the limitations of the instrumentation, this study did not investigate the performance of CsPbBr_3_ and CsPbBr_3_/aminopropyllsobutyl POSS under high-energy X-rays (e.g., X-ray energy > 60 keV). Future work should aim to overcome these limitations and conduct detailed studies on the performance of perovskite scintillators under high-energy X-rays. This progression will not only deepen our understanding of perovskite properties but also expand perovskite practical applications across various scientific and industrial domains.

## Resource availability

### Lead contact

Further information and requests for resources should be directed to and will be fulfilled by the lead author, Hai Liang (lianghai_ay/163.com).

### Materials availability

This study did not generate new unique reagents. All chemicals were obtained from commercial resources and used as received.

### Data and code availability

• Data: all data reported in this paper will be shared by the lead contact upon request.
• Code: this study does not generate a new code.
• Additional information: any additional information required to reanalyze the data reported in this study is available from the lead contact upon request.

## Acknowledgments

This work was supported by Anhui Province Key Research and Development Plan Project (no. 2022e07020066).

## Author contributions

H.L. and F.W. designed and conducted the experiments. R.X. and W.W. performed sample testing. H.L., F.W., and S.L. wrote the manuscript. P.D. and M.Y. helped write the manuscript. H.L. and S.L. supervised the research. All authors contributed to the general discussion.

## Declaration of interests

The authors declare no competing interests.

## STAR★Methods

### Key resources table

**Table:** 

REAGENT or RESOURCE	SOURCE	IDENTIFIER
**Chemicals, peptides, and recombinant proteins**		

CsBr	Macklin	7787-69-7
PbBr_2_	Macklin	2750-87-2
Oleic acid (OA)	Macklin	112-80-1
Oleylamine (Oam)	Macklin	112-90-3
aminopropyllsobutyl POSS	Macklin	65435-64-7
styrene ethylene butylene styrene (SEBS)	Aladdin	24937-78-8

**Software and algorithms**		

ImageJ	National Institutes of Health (NIH) image	imagej.nih.gov/ij/
DigitalMicrograp	Gatan, Inc.	www.gatan.com
Origin	OriginLab	https://www.originlab.com/

### Experimental model and study participant details

This study does not use experimental methods typical in the life sciences.

### Method DETAILs

#### Synthesis of CsPbBr_3_/aminopropyllsobutyl POSS nanocomposites

The CsPbBr_3_ NCs were synthesized by a hot-injection method. In a typical synthesis method: CsBr (0.052 g) and PbBr_2_ (0.089 g) powder were dissolved in 5 mL DMF with vigorously stirring, after adding 0.09 mL OA and 0.09 mL QAm, the mixed solution was heated between 85°C to dissolved all the powders. Then, 0.2 mL the hot mixed solution was injected into 5 mL toluene at room temperature. For the synthesis of CsPbBr_3_/aminopropyllsobutyl POSS nanocomposites, different amount of aminopropyllsobutyl POSS are pre-dissolved in toluene (0.05, 0.1 and 0.15 g/mL, respectively). 0.2 mL the hot CsBr: PbBr DMF solution are injected into the aminopropyllsobutyl POSS toluene solution, waiting for the CsPbBr_3_/aminopropyllsobutyl POSS nanocomposites growth.

#### Preparation of perovskite/SEBS film

5 mL CsPbBr_3_ NCs or CsPbBr_3_/aminopropyllsobutyl POSS nanocomposites solution were centrifuged and dispersed in 2 mL of toluene with 0.3 g of SEBS pre-dissolved. The mixed solution was injected homogeneously into a 20 × 20 mm Teflon mold. After waiting the toluene evaporated entirely, and the CsPbBr_3_ NCs or CsPbBr_3_/aminopropyllsobutyl POSS nanocomposites SEBS scintillator film were achieved and removed from the mold.

#### Characterization

The morphology of the synthesized CsPbBr_3_ NCs and CsPbBr_3_/aminopropyllsobutyl POSS nanocomposites was characterized using a transmission electron microscope (JEOL, JEM-2100). The powder X-ray diffraction (XRD) patterns of the obtained products were measured using an X-ray diffractometer (Corporation, SmartLab 9kw). The absorption spectra were obtained with a UV-Vis spectrophotometer (PerkinElmer, Lambda 750S). PL spectra were recorded by a spectrofluorometer (HITACHI, F2500).

#### X-Ray imaging

The X-ray source used in this study was a TD-3500 X-ray diffractometer (target material: Cu, tube voltage: 2–40 kV, tube current: 2–30 mA), manufactured by Dandong Tongda Industry Co., Ltd. Firstly, the X-rays penetrated the imaging object and were absorbed by the perovskite scintillator screen. The resulting image was then reflected by a mirror and captured by a CCD camera. Subsequently, the computer, connected to the CCD camera, processed the image using specialized software. The inclusion of a mirror in the optical path was crucial to prevent X-ray interference with the CCD camera.

### Quantification and statistical analysis

The size distribution of nanocomposites is analyzed by ImageJ software. The data analysis and plotting in the article were implemented using Origin software.
