# Supplementary material for: In-situ synthesized perovskite/polyhedral oligomeric silsesquioxane nanocomposites for robust X-ray imaging
Source: iScience. 2024 Sep 14;27(10):110951. doi: 10.1016/j.isci.2024.110951 (PMC11467670; doi:10.1016/j.isci.2024.110951)
Supplement: Document S1. Figures S1–S11 [file mmc1.pdf]

iScience, Volume *27*

Supplemental information

In-situ synthesized perovskite/polyhedral oligomeric silsesquioxane nanocomposites for robust X-ray imaging

Hai Liang, Fan Wu, Runan Xia, Wei Wu, Siqi Li, Panpan Di, and Miao Yang

Document S1. Figures S1–S11

Supplemental Information

In-situ synthesized perovskite/polyhedral oligomeric silsesquioxane nanocomposites for robust X-ray imaging

Hai Liang[#1], Fan Wu[#2], Runan Xia[1], Wei Wu[1], Siqi Li[2], Panpan Di[1], Miao Yang*[1]

1.Department of Pharmacy, The People's Hospital of Bozhou, Bozhou, Anhui Province, P. R. China

2.School of Physics and Optoelectronic Engineering, Anhui University, Hefei, 230601, Anhui, P. R. China

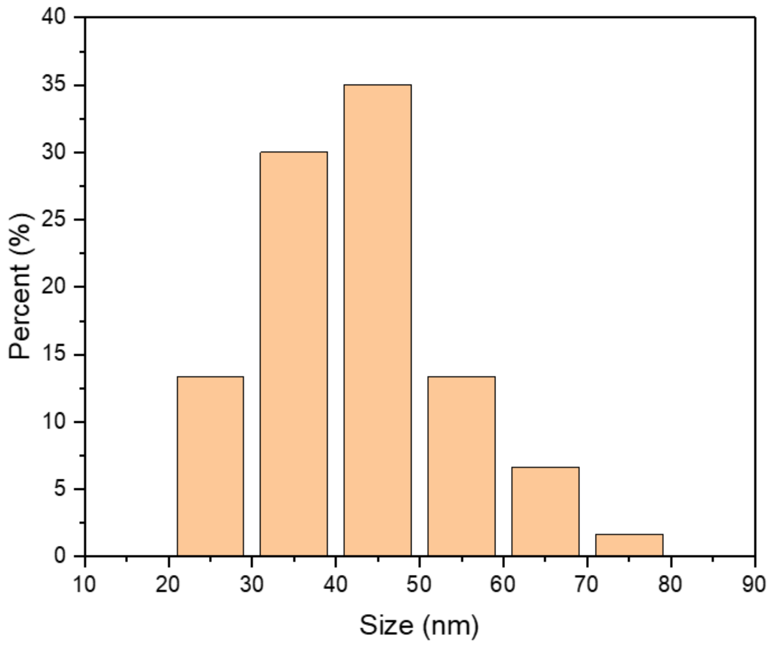

Figure S1 The size distribution of $CsPbBr_3$ nanocrystals. Related to Figure 1.

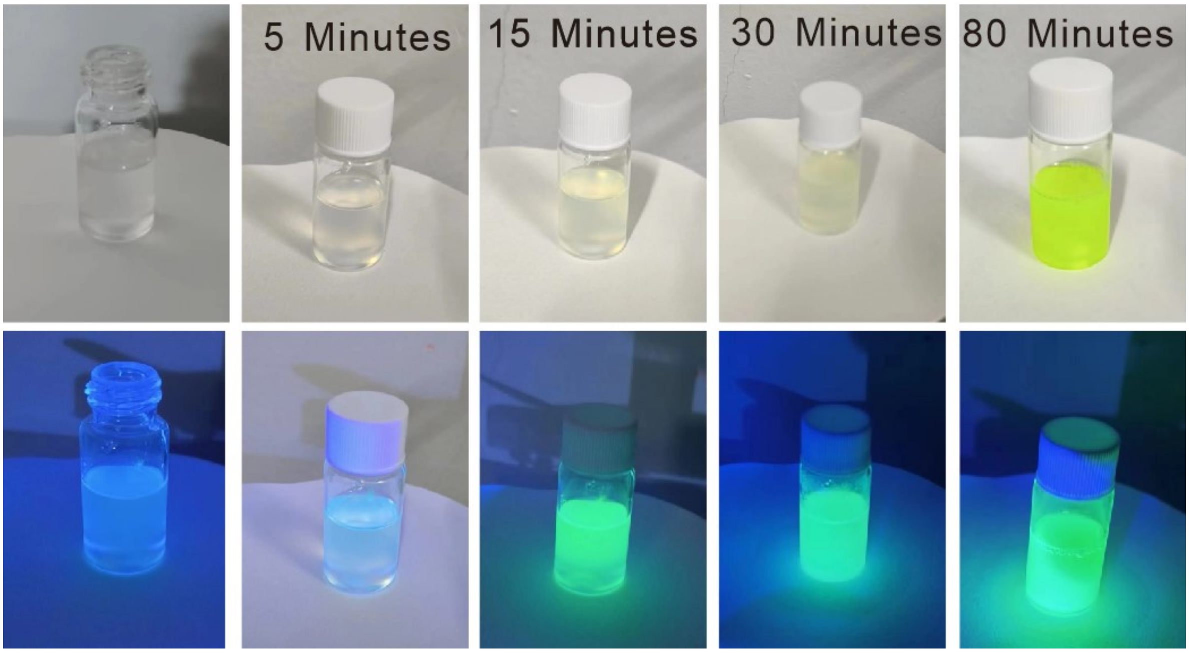

Figure S2 The pictures of $CsPbBr_3$/aminopropylIsobutyl POSS nanocomposites solution under ambient light and UV light at different stages. Related to Figure 1.

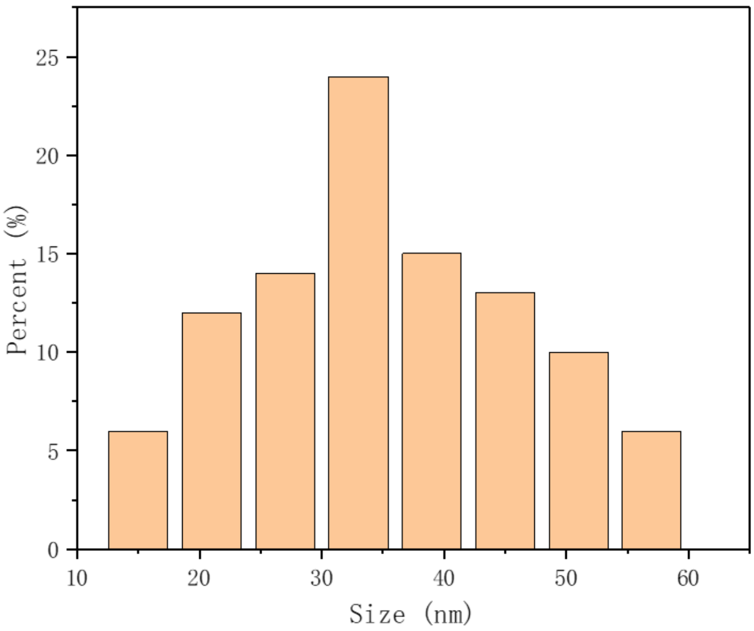

Figure S3 The size distribution of $CsPbBr_3$ nanocrystals inside the aminopropylIsobutyl POSS matrix. Related to Figure 1.

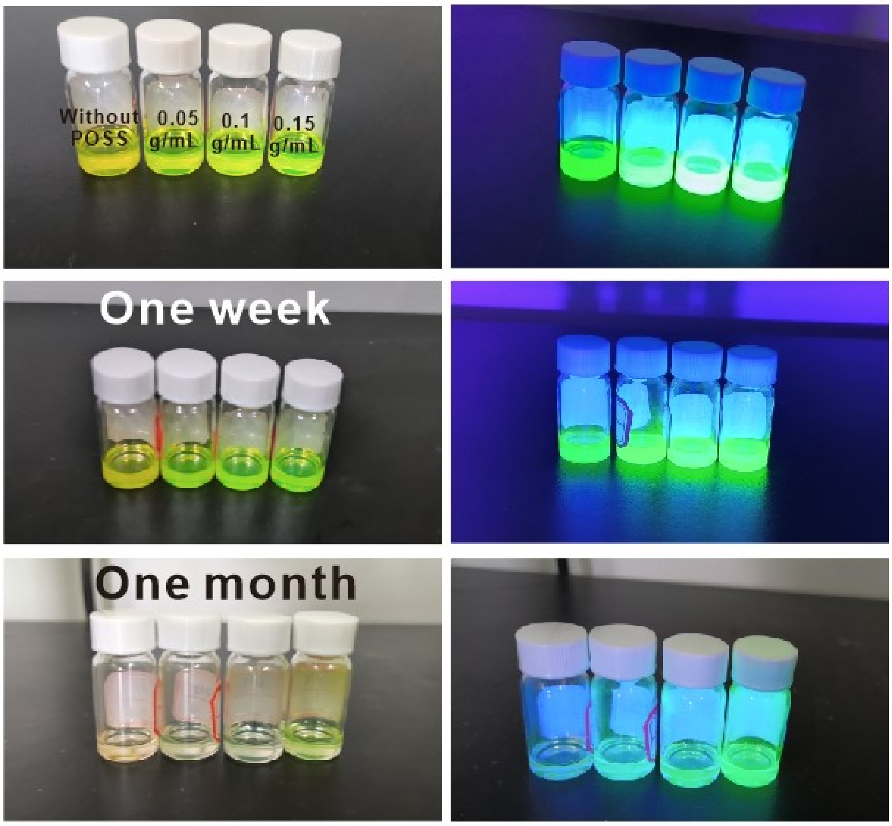

Figure S4 Comparison of long-term storage effect of $CsPbBr_3$/aminopropylIsobutyl POSS nanocomposites and $CsPbBr_3$ nanocrystals. Related to Figure 1.

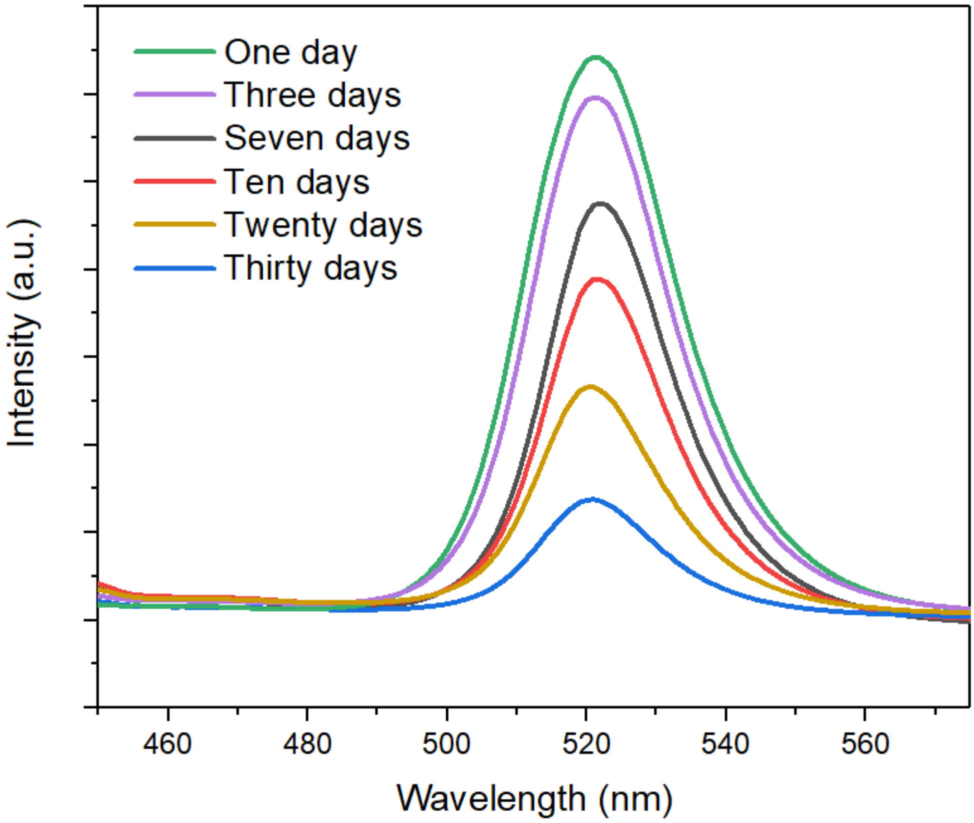

Figure S5 The recorded PL intensity of $CsPbBr_3$/aminopropylIsobutyl POSS nanocomposites after long-term storage. Related to Figure 2.

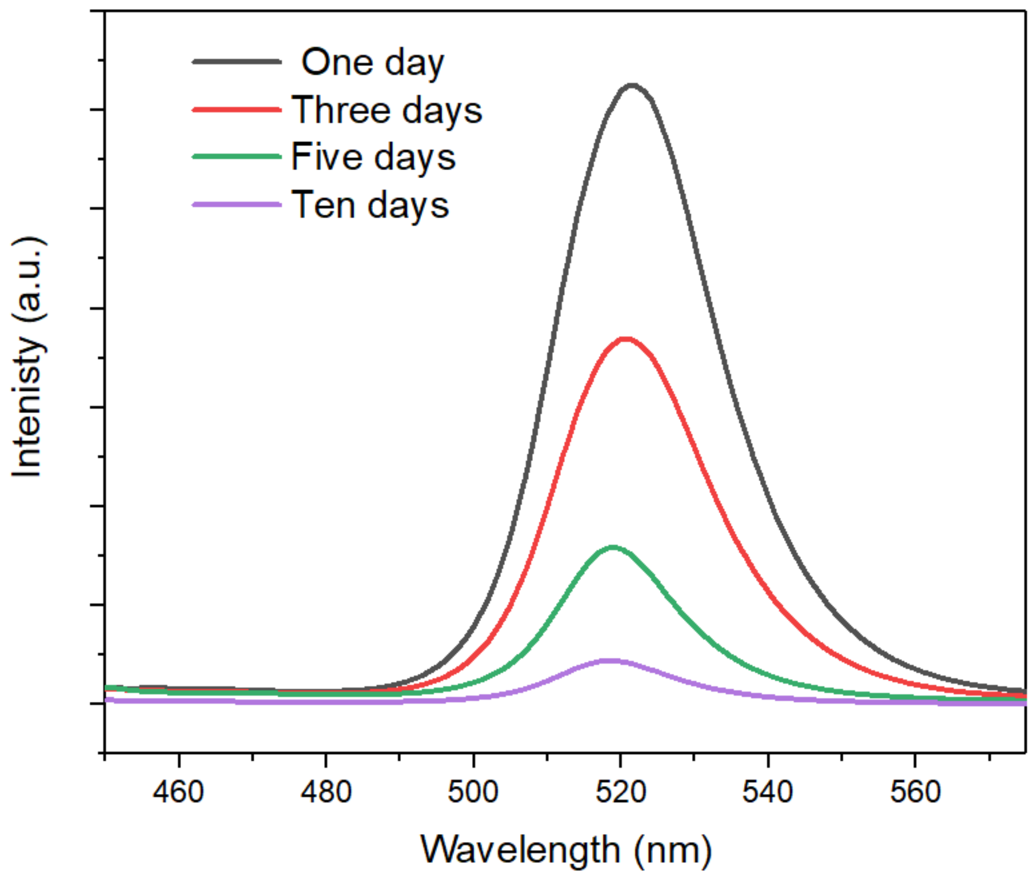

Figure S6 The recorded PL intensity of $CsPbBr_3$ nanocrystals after long-term storage. Related to Figure 2.

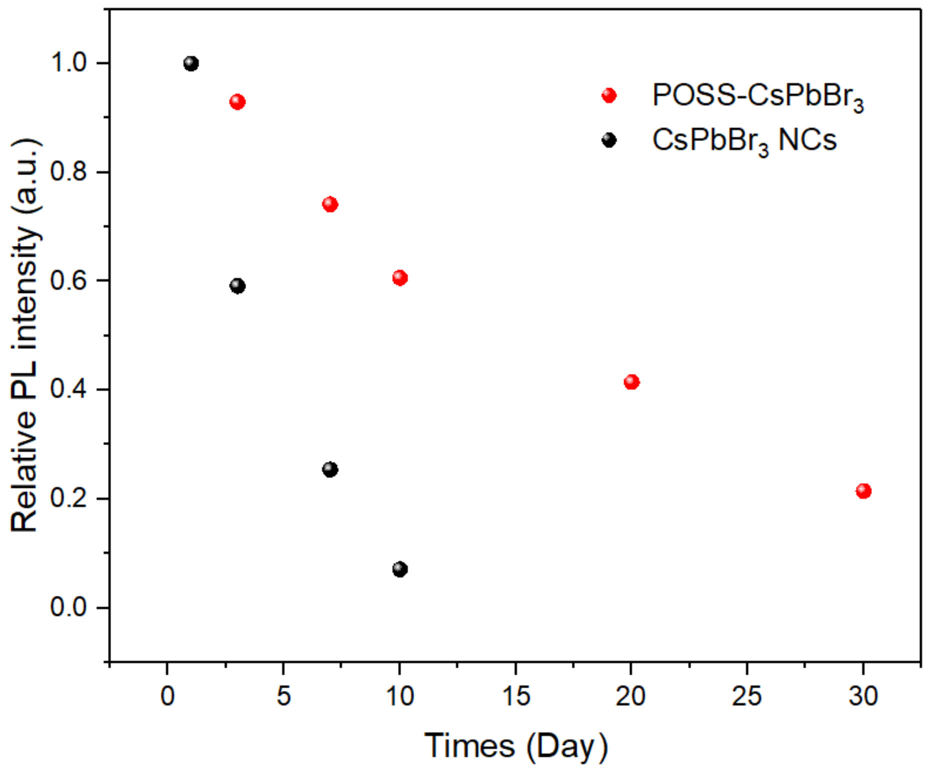

Figure S7 The emission comparison of $CsPbBr_3$/aminopropyllsobutyl POSS nanocomposites and $CsPbBr_3$ nanocrystals after long-term storage. Related to Figure 2.

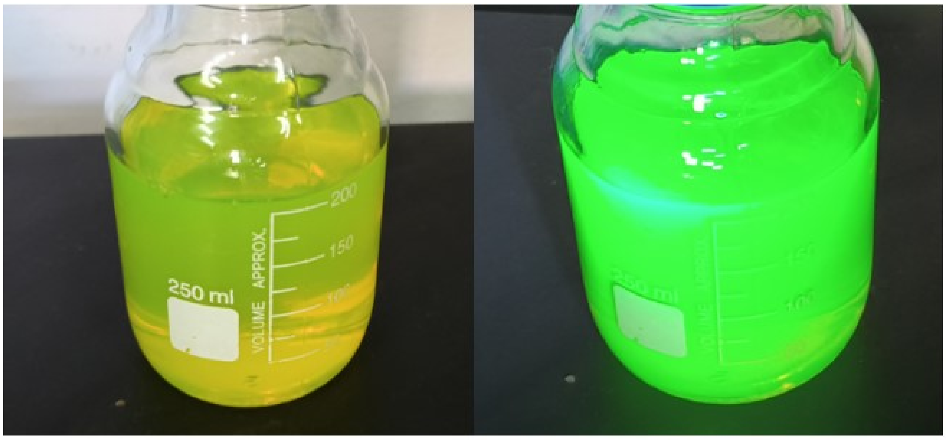

Figure S8 One step of large-scale preparation of $CsPbBr_3$/aminopropylIsobutyl POSS nanocomposites. Related to Figure 2.

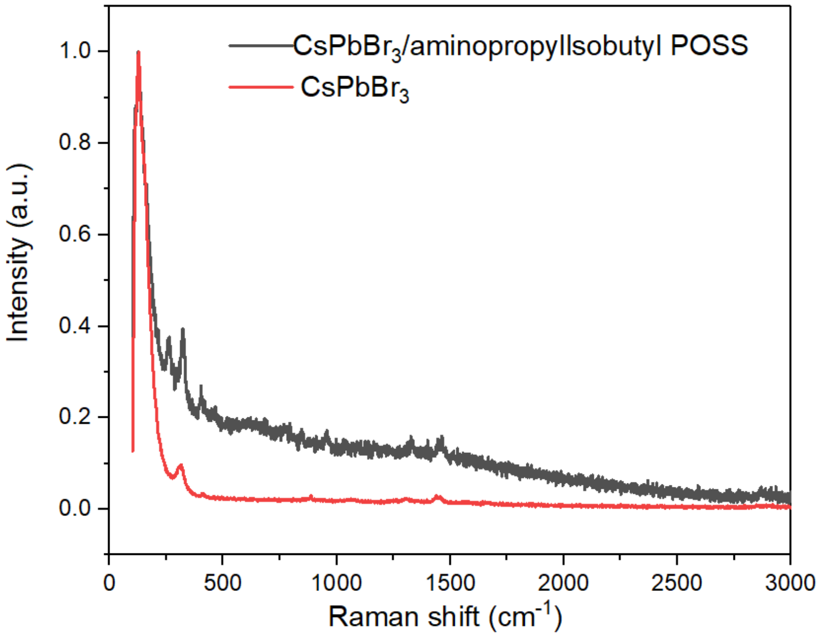

Figure S9 The Raman spectra of $CsPbBr_3$ NCs and $CsPbBr_3$/aminopropylIsobutyl POSS nanocomposites using a 785 nm laser. Related to Figure 3.

As shown in the Raman spectra, compared to pure perovskite, a series of additional peaks, 263 cm^{-1}, 322 cm^{-1}, 465 cm^{-1}, 956 cm^{-1}, are evident. These peaks

correspond to the vibration of Si-O-Si in aminopropyllsobutyl POSS.

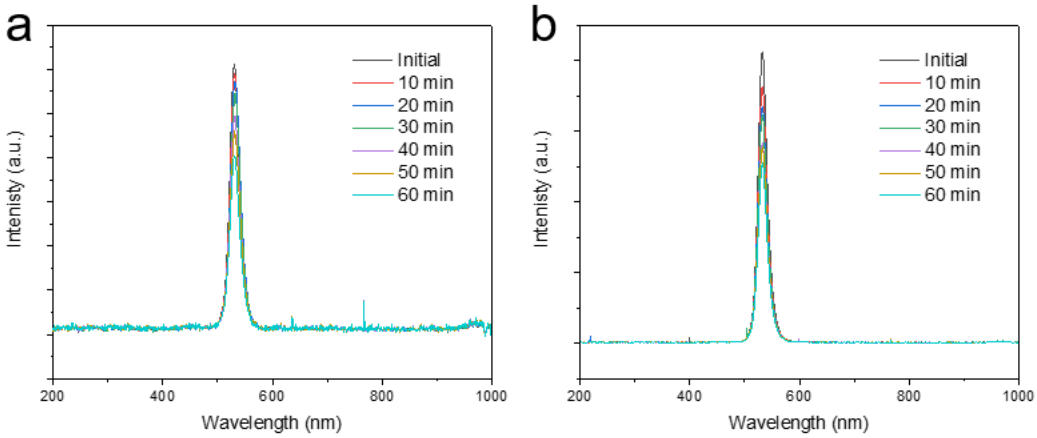

Figure S10 The recorded RL of $CsPbBr_3$/aminopropyllsobutyl POSS and $CsPbBr_3$ nanocrystals under continuous irradiation. Related to Figure 4.

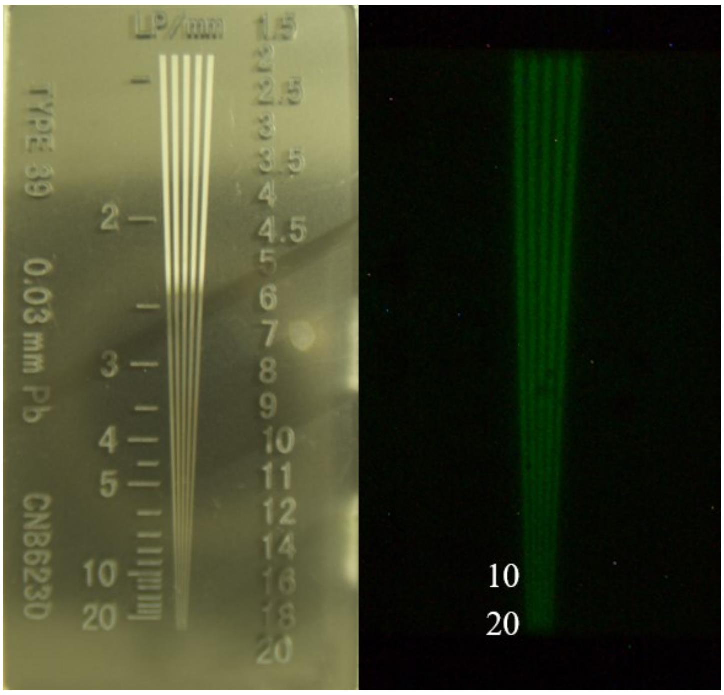

Figure S11 X-ray images of a local area of a standard X-ray test scale using $CsPbBr_3$/aminopropyllsobutyl POSS nanocomposites film. Related to Figure 5.
